# Bioactive Compounds in Food Waste: A Review on the Transformation of Food Waste to Animal Feed

**DOI:** 10.3390/foods9030291

**Published:** 2020-03-05

**Authors:** Alexandros Georganas, Elisavet Giamouri, Athanasios C. Pappas, George Papadomichelakis, Fenia Galliou, Thrassyvoulos Manios, Eleni Tsiplakou, Kostas Fegeros, George Zervas

**Affiliations:** 1 Laboratory of Nutritional Physiology and Feeding, Faculty of Animal Science, Agricultural University of Athens, 11855 Athens, Greece; stud213016@aua.gr (A.G.); egiamouri@aua.gr (E.G.); gpapad@aua.gr (G.P.); eltsiplakou@aua.gr (E.T.); cfeg@aua.gr (K.F.); gzervas@aua.gr (G.Z.); 2 Department of Agriculture, Hellenic Mediterranean University, 71410 Heraklion, Crete, Greece; fgalliou@hmu.gr (F.G.); tmanios@hmu.gr (T.M.)

**Keywords:** amino acids, bioactive compounds, broilers, catering waste, fatty acids, feed, food waste, layers, minerals, restaurant waste, swine, vitamins

## Abstract

Bioactive compounds are substances which are present in foods in small amounts and have the ability to provide health benefits. Bioactive compounds include but are not limited to long-chain polyunsaturated fatty acids, vitamins, carotenoids, peptides, and polyphenols. The aim of the present study is to review literature for potential bioactive compounds present in food waste material and discuss the transformation of food waste to animal feed under the perspective that usage of food waste, rather than disposal, may tackle food insecurity and provide health benefits. Finally, applications in poultry and swine nutrition, with emphasis on the presence of fatty acids on food waste material, are discussed.

## 1. Introduction

Advances in the agri-food sector are necessary in order to promote global food security and nutrition, environmental sustainability, and to benefit global food production by reducing production costs [1,2]. According to the 2019 World Population Prospects of the United Nations, the world’s population is estimated at 7.7 billion and is projected to increase to 9.7 billion by the middle of this century [3]. More than 820 million people suffered from hunger in 2018, and about 2 billion people undergo moderate or severe food insecurity globally [2]. The challenge to meet the nutritional demands of the growing population should take also into account cases of undernourishment or micronutrient deficiency. On the other side, in other parts of the world, obesity may become a big health issue by 2025 [4]. At the same time, dietary habits may alter since processed food products are becoming an increasing part in human nutrition, which will result in increasing consumption of sugar, oils, and fats compared to consumption of staples and protein [4]. An increase in demand for animal products, such as meat, is projected to take place until 2050 [5], which will, in turn, result in an increasing pressure on the food market to meet consumer demand [6]. In order to align with demand, meat production is projected to double at an amount of 455 million tons by 2050 [7]. In the last decades, the poultry sector has grown rapidly in comparison with the other livestock production sectors [7], and many people in the world consume chicken meat and eggs to meet a large proportion of their protein needs [8]. However, it is very likely that food production needed to correspond with consumer demand will be limited due to finite natural resources [5]. Agricultural land, which is available for food production, may not expand and it is even possible for land to be less available for that purpose [5].

Concurrently, one-third of the total food produced is lost or wasted globally, an amount of food equal to 1.3 billion tons [9]. Food losses that arise at the final sectors of the food supply chain, namely, retail and final consumption, are referred to as “food waste”, whereas food losses occurring during production, post-harvest and processing stages in the food supply chain are regarded as “food loss” [9,10]. Food waste accounts for a substantial quantity of wasted resources [11], most notably, the nutrients present. Food waste relates to wasted food nutrients of premium quality intended for human consumption [12] and, therefore, it usually has a good nutritional value [13]. A huge portion of food waste, which includes edible and inedible parts of food estimated at 88 million tons, is generated in the European Union (EU) every year [14]. Of the food which is lost or wasted in the EU, 12% derived from food services, 53% from households, 5% from wholesale and retail sectors, and the remaining 30% derived from the production and processing sectors [14]. On the contrary, in developing countries, food losses that occur at the early and middle stages of the food supply chain outnumber food waste at later stages, namely, the retail sector and final consumption [12], indicating that food loss occurs mainly during production, handling, and storage [12]. Sources of food losses at the initial stages of the food supply chain include but are not limited to losses during harvesting (edible crops left in the field, crops damaged during harvesting), losses during transportation and storage. In latter parts of the supply chain, losses may occur during processing, product evaluation, packaging, and marketing. Sources of food waste at the final stages are households, food services (restaurants, hospitality sector, cafes), and institutions (education institutions, prisons, and hospitals) [15]. Examples of food waste include kitchen waste that is not served for consumption and plate waste that includes food discarded after food is being served [16]. Although the main effort to reduce food waste should be on prevention, its re-entrance in the food supply chain is a prerequisite for putting into effect the principles of the circular economy; the latter considers waste as resources [17]. Food waste material generated at the latter parts of the food supply chain, i.e., retail, food service, and consumption at home, has been extensively studied compared to waste material generated at an earlier stage of the chain. However, it was reported that limited research exists on the chemical composition and variation between food waste types [18].

Increasing demand for animal products is projected to lead to further demand for feed, especially from coarse grains, such as maize, and protein meals by 2025 [4]. Conventional poultry and swine diets are mainly based on maize and soybean as energy and protein sources, respectively [19,20,21], while a large part of the world population hinges on maize as a staple food, especially in developing countries [22]. Food waste could be a substitute for part of cereal grains and plant protein sources used in animal nutrition, which would alleviate food competition between humans and animals [23]. Additionally, feed costs represent a significant component in meat production and it impacts financial gains [24]. Feed costs range from 55% to 72% of total pig production costs in the EU [25,26] and from 55% to 75% of the total poultry production costs [24]. Therefore, production costs could be lessened with the use of food waste due to its lower cost compared to conventional feeds [26].

Besides its magnitude in combating food insecurity, it has the potential to promote health benefits as food waste may contain valuable substances, such as bioactive compounds. From the perspective of human nutrition, the transformation of food waste to animal feed has a twofold potential: tackle food insecurity and provide health benefits. The aim of the present study is to review literature for potential bioactive compounds present in food waste material and discuss the transformation of food waste to animal feed.

## 2. Literature Review: Bioactive Compounds in Food Waste

Loss and waste of highly perishable foods, such as fruits, vegetables, and animal-based products, lead to losses of important nutrients [2,9]. In general, food waste has a good nutritional value [27] because constituents present in the food waste are of premium quality since they were intended for human consumption [28]. However, nutrient variability of food waste is one of the main issues that complicate its incorporation in animal diets [13]. The factors that determine the fluctuation of the nutritional composition of food waste are its source [27] and other factors in relation to consumers, such as their age profile, ethnic origin, and dietary habits [16,28,29,30].

Food waste has typically high moisture content [27], between 50% and 85% [16], which reduces shelf-life and makes food waste collection and its incorporation in animal diets difficult [31]. It has been suggested that the heat treatment needed to produce a safe food waste product is adequate for the reduction of moisture content [29].

Bioactive compounds are substances that are present in food [32] in small amounts [33] and that have the ability to provide health benefits [34]. More specifically, bioactive compounds positively affect the human organism or specific tissues or cells [32]. It has been demonstrated that a variety of compounds present in products of plant and animal origin have beneficial effects on human health [32]. Bioactive compounds include but are not limited to long-chain polyunsaturated fatty acids (PUFA) [32,35], vitamins, carotenoids, peptides, and polyphenols [32]. Long-chain PUFA of major significance include eicosapentaenoic acid (EPA) [35], docosahexaenoic acid (DHA), and arachidonic acid [35]. Meat and other meat products have appreciable content of bioactive compounds [36], and thus, food waste products originating from meat or fish may also contain bioactive compounds. In recent years, there has been growing interest among consumers and producers of contemporary livestock production in the production of healthier meat and derived products [36]. Therefore, their incorporation into animal diets has the potential to lead to the production of value-added products [18].

### 2.1. Amino Acids

In poultry and swine nutrition, both quantity and quality of dietary protein are significant [37]. Regarding the quantity of protein in food waste, it has been reported that the crude protein (CP) of restaurant waste ranges from 15% to 23% on a dry basis, as calculated in four different studies [37]. In a study conducted by Fung et al. [38], CP in food waste from a University residential dining hall was approximately 18.9% on a dry basis, which was higher than that of corn but lower than that of dehulled, solvent-extracted soybean meal (CP 47.2%). Similar to the previous studies was the CP in restaurant (kitchen) food waste and plate waste analyzed in the experiment of Myer et al. [16], which ranged from 18% to 20% on a dry basis. Furthermore, CP content in leftover food was equal to 22% on a dry basis (CP 20.62%, dry matter 93.70%), as reported by Cho et al. [39]. Kwak and Kang [40] found the same result of CP (22.0%) on a dry basis in restaurant waste. Higher CP content in restaurant waste was reported by Chae et al. [41] to be about 25% on a dry basis, and in food waste from households and the food service sector in another study by Castrica et al. [28] to be approximately equal to 27.6% on a dry basis (or CP 24% on a wet basis as reported). Garcia et al. [29] found that CP in restaurant waste was also 27.5% on average on a dry basis. An even higher CP content in leftover food from a restaurant and a hotel was found by Asar et al. [42] to be approximately equal to 31.3% on a dry basis (or CP 17.4% on a wet basis as reported; Table 1).

When corn and soybean meal is used, lysine and methionine are limiting amino acids in swine [38] and poultry diets [43]. Fung et al. [38] analyzed the amino acid profile of food waste deriving from a University residential dining hall. Food waste presented a higher content of lysine, methionine, threonine, and tryptophan compared to corn, whereas it was lower than that of soybean meal [38]. Cho et al. [39] found a lower methionine and lysine content in leftover food compared to the food waste of the previous study. In another experiment, Choe et al. [44] analyzed the amino acid profile of restaurant waste and a standard conventional diet for growing-finishing pigs and found that methionine, cysteine, and lysine were quite similar between the two diets, while threonine and valine were higher in food waste compared to the conventional diet used in this study. However, in a study conducted by Chae et al. [41], the content of most essential amino acids, such as methionine and lysine, of food waste collected from restaurants and apartment complex areas was considerably lower than that of a corn and soybean mix (60%:40% ratio). Furthermore, the digestibility of amino acids present in food waste should be examined on animals prior to feeding due to the processing and heating of food waste [38]. Animal products such as fish, eggs, and milk contain a higher degree methionine and lysine compared to the protein supplements of plant-derived materials [45]. Lipinski et al. [12] reported that the quantity of meat, fish, and seafood, which are lost or wasted calculated on the caloric content basis, account for 25% (375 trillion kcal) of the total food lost or wasted (1.5 quadrillion kcal). Above all, food waste can be a good source of protein in animal nutrition; however, it is variable.

### 2.2. Minerals

Mineral content (calculated as ash) in restaurant waste ranged from 3% to 6% on a dry basis in four different studies, as reported by Myer et al. [37]. Similar results showed the experiment conducted by Myer et al. [16], in which the mineral content in restaurant waste was 5–6% on a dry basis [37]. Fung et al. [38] found that ash was approximately 5.01% in food waste from a dining university hall, which was consistent with the results previously mentioned. However, substantially higher ash content in food waste was reported by Kwak and Kang [40] (originating from restaurants), Castrica et al. [28] (deriving from commercial and residential areas), and Asar et al. [42] (originating from restaurant and hotel) equal to 12.6%, 14.56%, and 14.75% on a dry basis, respectively.

Food waste might have higher salt content compared to conventional feeds [37]. This is supported by the results of the experiments conducted by Myer et al. [16], and Chae et al. [41], in which the salt content was about 2.0% to 2.5% in restaurant waste and about 3.28% on a dry basis in food waste deriving from restaurant and apartment complex areas, respectively. The latter mentioned salt content of food waste was much higher than that of a corn and soybean mix (60%:40% ratio), as reported by Chae et al. [41]. In line with these results was the salt content of restaurant waste analyzed by Choe et al. [44], which was significantly higher compared to a standard conventional diet for growing-finishing pigs. Food waste quantity in pig [37] and poultry diets could be restricted due to the high salt content if it exceeds the upper limit that ensures the welfare and good growth performance of the animals. Salt rations of diets above tolerance levels may lead to salt poisoning of pigs and poultry. Moreover, elevated salt content in pig diets may bring about a soft texture of pork, which is subject to rancidity in a shorter amount of time [44]. However, food waste could substitute salt supplementation [37] if incorporated in the diet properly.

Choe et al. [44] compared the phosphorus and calcium contents in restaurant waste and a standard conventional diet for growing-finishing pigs and found that the phosphorus and calcium contents were similar between the two diets. Fung et al. [38] reported that food waste from a university dining hall contained 0.04% to 0.46% calcium on a dry basis, which is similar to some extent to that of soybean meal (Ca 0.27–0.47%, with CP 47.2%) and can be much higher than the calcium content (0.01–0.03% on a dry basis) in corn. In the previously mentioned study, phosphorus content in food waste (0.23–0.37%) was lower than that of soybean meal (0.70–0.88%) but similar to the phosphorus content of corn (0.24–0.34%) on a dry basis [38]. Higher contents of calcium (0.5–0.8%) and phosphorus (0.3–0.8%) on a dry basis in food waste in comparison with the above-mentioned study were found by Myer et al. [16], which was higher compared to soybean meal (Ca 0.27–0.47%) and corn (Ca 0.01–0.03%) in the case of calcium, and can be similar or intermediate between soybean meal (P 0.70–0.88%) and corn (P 0.24–0.34%) in the case of phosphorus. Even higher contents of these macronutrients in restaurant waste were found by Garcia et al. [35], which ranged from 0.42% to 1.7% calcium, and 0.81% to 2.07% phosphorus on a dry basis. Castrica et al. [28] found similar calcium content (1.09–1.25%) in food waste, but lower phosphorus content (0.16–0.30%) on a dry basis. Castrica et al. [28] also found that potassium content in food waste originating from commercial and residential areas ranged from 0.56% to 0.76%, and magnesium content ranged from 0.1% to 0.2% on a dry basis (Table 1). Furthermore, according to our knowledge, there is no available data regarding the bioavailability of phosphorus in food waste. This is a significant matter in feedstuffs, and further research is necessary to facilitate the inclusion of food waste in animal diets [18]. It can be concluded that, regarding the minerals present in food waste, there is variability in the content, lack of data on the bioavailability of phosphorus, and reports that high content of salt may be present.

### 2.3. Fatty Acids

In general, food waste contains relatively high ether extract (EE) [38]. Myer et al. [37] reported that EE content in restaurant waste ranged from 17% to 24% on a dry basis, as obtained from four different studies. In a study conducted by Chae et al. [41], EE content in food waste from restaurants and apartment complex areas was about 17.3% on a dry basis, and thus it was consistent with these results. Similar to the previous results was the EE in restaurant waste reported by Kwak and Kang [40], which was equal to 23.9% on a dry basis. Fung et al. [38] found lower EE content in food waste from a dining university hall equaled to 13.58% on a dry basis, while Cho et al. [39] found EE content in leftover food equal to 10.66% on a dry basis (EE 9.99%, dry matter 93.70%). An even lower EE content in food waste from commercial and residential locations was reported by Castrica et al. [28], equaled to 9.12% on a dry basis (EE 7.94% on a wet basis as reported), whereas higher EE content in leftover food from a restaurant and a hotel was estimated at 26.08% on a dry basis (EE 14.5% on a wet basis as reported) by Asar et al. [42]. The latter was similar with the result of an experiment conducted by Myer et al. [16], in which EE content in restaurant waste was equal to 24–26% on a dry basis, while restaurant waste examined by Garcia et al. [29] had 28.8% EE on a dry basis. Taking into consideration that food waste has relatively high CP and EE content, while it is inexpensive, and that protein and energy stored in fat are feed constituents that make up substantial feed costs, food waste may be a good source of protein and fat [38] as long as no quality degradations occur.

PUFA content in food waste can be worthy of comparison with corn oil, though it is contingent on the fats and oils present in food waste [38]. Fung et al. [38] found that food waste that contained 13.58% EE from a university dining hall had appreciable content of arachidonic acid (on average 0.20% of EE), linoleic acid (29.31% of EE), and linolenic acid (3.82% of EE). In another study conducted by Choe et al. [44], PUFA content and polyunsaturated fatty acid/saturated fatty acid ratio (PUFA/SFA ratio) in restaurant waste were significantly higher than that of the conventional diet used in swine nutrition in this study. The PUFA in restaurant waste investigated in the aforementioned study were linoleic acid, linolenic acid, and DHA, and the n-6/n-3 ratio was significantly lower in restaurant waste compared to the conventional diet [44]. Furthermore, saturated and monounsaturated fatty acid (MUFA) contents were significantly higher in the conventional diet in comparison to the restaurant waste used in the study conducted by Choe et al. [44]. The fatty acid profile of food waste deriving from commercial and residential locations was also examined by Castrica et al. [28]. PUFA/SFA ratio in the food wastes was approximately 0.78, and the n-6/n-3 fatty acid ratio was about 7.94. In the above-mentioned study, oleic acid (30.63% of EE) in food wastes was quantitatively the highest among the other fatty acids. Moreover, linoleic acid and linolenic acid content were 25.5% and 3.03% of the total fatty acids, respectively [28] (Table 1).

The nutritional value of food waste generated from food services depends on the type of food operation. In general, food waste that derives from sumptuous hotels and restaurants has higher nutritional value in comparison to that of fast food enterprises [47]. Fats and oils of food waste are not an exception as processing methods of food preparation like frying may have adverse effects on the quality of food. Long exposure of food to heat results in thermo-oxidative degradation of fatty acids and the formation of an off-flavor [48]. Furthermore, exposure of fatty acids to high temperatures provokes chemical reactions in the products (e.g., trans fatty acids), which are undesirable [48].

### 2.4. Vitamins

There has not been conducted much research on the vitamin content of food waste with regard to animal nutrition. In an early study, Kornegay et al. [46] investigated the vitamin content of restaurant and hotel waste. In these types of food waste, the thiamine and niacin concentrations were adequate to meet the nutritional requirements for swine, while the pantothenic acid concentration was deficient. Kornegay et al. [46] stated that these results were different compared to other early studies in which niacin and pantothenic acid concentrations of restaurant and hotel waste were higher.

It has been demonstrated earlier in the past century that animal proteins in poultry nutrition had better results compared to plant proteins. Partially, this is attributed to the higher B-complex vitamins, especially riboflavin, content in dried skimmed milk and whey, and to vitamin B12, which is present in all animal products, but not in plants [45]. Therefore, food waste which contains animal products such as the previously mentioned may have considerable content of vitamins important in swine and poultry nutrition, which is much higher than in plant-origin feedstuffs.

## 3. Food Waste Conversion into Animal Feed

### 3.1. Legal Framework Regarding Food Waste Utilization in Animal Feed

Food waste disposal has big consequences for the environment, and therefore, a “waste management hierarchy” has been adopted, defining the most and the least preferable disposal options [49]. The best way to face the food waste disposal problem is the prevention of wasting food, and secondly, the re-use and redistribution of food. Moreover, recycling food waste in animal nutrition and composting, anaerobic digestion, and landfill disposal are less preferable options in order of priority [49]. Other uses of food waste include industrial treatments for the production of biofuels and biopolymers [50].

The European directive on waste sets a goal to limit the municipal waste that ends up in landfills to 10% by 2030. Furthermore, it promotes the implementation of new measures that increase the prevention and re-use of food waste [51]. The Food and Agriculture Organization (FAO) of the United Nations has set 17 goals for promoting sustainable development. The goal of tackling food insecurity and fostering sustainable agriculture can be boosted by applying feeding strategies and utilizing feedstuffs that are capable of increasing livestock productivity and that have a lower environmental impact in comparison to conventional animal production [52]. The use of food waste in animal nutrition has the potential to improve food security and contribute to the reduction of the environmental impact of the agri-food system and the production costs of animal products.

In the EU, the use of catering waste in animal feed, except for fur animals, was prohibited in 2002 [53], and the prohibition is still in force since then [54]. This ban was a consecutive action after the foot-and-mouth disease outbreak that took place in the United Kingdom (UK) in 2001, due to the feeding of uncooked food waste to swine [26,55]. In a wider context, the emergence of foot-and-mouth disease, bovine spongiform encephalopathy, and the presence of dioxins in feedstuffs led to the ban of the use of animal by-products and derived products in farm animal nutrition [54]. Similar outbreaks of diseases, such as vesicular exanthema, classical swine fever, and trichinosis have been associated with the feeding of uncooked garbage in the United States (US), of which the vesicular exanthema outbreak resulted in the prohibition of the same practice in this country in the 1950s [56]. It should be noted that garbage includes not only food waste deriving from the handling, preparation, cooking, or consumption of food, but it also includes animal materials, such as meat [57].

Currently, under the EU legislation being in force, a small proportion of the generated food waste can be utilized in animal nutrition [26,58]. According to the European Former Foodstuff Processors Association (EFFPA) [59], food waste appropriate for use as animal feed represents only 5 million tons of food waste, which is a small proportion compared to the total food waste generated in the EU [26]. Of the 5 million tons of food waste that can be utilized in animal nutrition, 3.5 million tons are used for this purpose every year [26,59].

Safety hazards that are associated with the incorporation of food waste in animal diets are biological, chemical, and physical hazards. Many swine diseases have been related to the feeding of uncooked food waste and garbage to swine. African swine fever is usually linked to garbage feeding that comes from international airports [60]. Catering waste that originates from means of transport that operate internationally is banned for use in animal nutrition in the EU [54]. Classical swine fever and swine vesicular diseases, which also affect swine, can be spread through the feeding of infected pork products to swine [61,62], and thus food waste may be a source of infection in the case it contains such products. Concerns about the application of food waste recycling in poultry nutrition in the UK include the prevention of the spread of diseases such as avian influenza and Newcastle disease [63]. Furthermore, infectious organisms of public health significance that may be linked to feeding food waste to livestock include *Salmonella*, *Campylobacter*, *Mycobacterium*, *Trichinella*, *Toxoplasma* [56], and *Clostridium* [64]. Chemical hazards of food waste that are necessary to be assessed include antibiotics [65], mycotoxins, pesticides, and heavy metals, such as Pb, Cd, As, and Hg [28]. Fung et al. reported that biogenic amines in food waste intended for animal feed should also be determined [38]. Biogenic amines may be present in high protein feeds and their production is a result of bacterial activity [38]. The presence of biogenic amines in animal diets may have negative effects on growth performance and result in toxicity in animals [38]. Furthermore, physical hazards include plastic, glass, metal, and other materials [28], which have to be separated from food waste [29].

Appropriate processing of food waste is fundamental in order to reduce the risk of animal-to-animal and animal-to-human disease transmission [56] by achieving adequate microbial reduction at a level that ensures its safety [65]. For this purpose, heat treatment plays a major role [29]. Castrica et al. [28] examined the safety of food waste that was transformed into animal feed after proper treatment based on a US case study, according to the EU legal framework, and reported that food waste was consistent with the EU legislation regarding the safety requirements of feed production. In another study, Chen et al. [66] assessed the safety of chicken meat fed on a dehydrated food waste product and found that in that particular waste product, the dioxin, organic chloride, agrochemical, and heavy metal concentrations were consistent with FDA (U.S. Food and Drug Administration) regulations. However, Garcia et al. [29] reported that restaurant waste, which was analyzed in their experiment, contained lead in concentrations higher than the maximum permitted by the EU legislation value, which may have been a result of contact with materials such as cans and piping [26,29]. Moreover, Garcia et al. [29] found, in the aforementioned experiment, increased content of furans in restaurant waste that make it unsuitable for livestock feed, while dioxin-like PCBs (polychlorinated biphenyls) content was higher than that of other foods.

In countries where feeding food waste to animals is permitted, specific treatment of food waste prior to feeding is mandatory. In the US, it is required that food waste is processed by means of heating treatment at 100 °C for 30 min [67]. In Japan, treated food waste intended for animal feeding is designated as “ecofeed”. For the production of “ecofeed”, cooking of raw materials that may contain uncooked meat is required at 70 °C for 30 min or at 80 °C for 3 min, while cooking of raw materials that do not contain uncooked meat is preferable. Drying and ensiling (fermentation with lactic acid bacteria) of food waste is also used for a greater shelf life of “ecofeed” [68]. Moreover, the legislation in the US [67] and Japan [68] determine rules on the collection, handling, storage, and transportation of food waste.

Anti-nutritional factors may also be present in food waste in the event that it contains components of plant origin, for instance, legume seeds, oilseeds, peels, leaves, root tubers, and grain [29]. Anti-nutritional factors, such as enzyme inhibitors, lectins, tannins, alkaloids, and oligosaccharides [29], are compounds the activity of which leads to the decrease of nutrient utilization and/or feed intake [69]. The inactivation of anti-nutritional factors can be achieved by processing methods such as dry and wet heating, soaking [29,70], and extrusion [70]. The applied processing treatment of food waste should take into account, besides the reduction of the moisture content and the microbial load, the inactivation of anti-nutritional factors [29]. Given the large quantities of valuable animal products that are wasted, future studies may reveal those practices that will minimize risks and allow the use of food waste of animal origin in animal feed.

Several technologies have been implemented in order to reduce the moisture content, the nutrient variability, and the microbial load of food waste, and inactivate the presence of anti-nutritional factors. Processing of food waste is a fundamental step prior to its utilization in animal nutrition as it may facilitate its incorporation in animal diets.

### 3.2. Transformation of Food Waste to Feed

Food waste should be processed in order to be incorporated into animal diets, as it presents some undesirable properties such as nutrient variability. The fluctuation of the nutrient composition of food waste can be reduced with proper measurements. Strict control of the source of food waste that is intended for use in animal nutrition and/or the supplementation of food waste during processing with other feedstuffs, with the aim to formulate a balanced diet, have been suggested [27].

The processing technologies that are available today can lead to the transformation of food waste to safe animal feed products, which have additional value and that contain nutrients of premium quality [18]. Processing treatments of food waste include cooking, extrusion, pelletizing, dehydration, ensiling [31], and probiotic treatment [71]. Of the aforementioned treatments, extrusion, pelletizing, and dehydration bring about greater shelf life of the final food waste product compared to cooking and ensiling [31]. In addition, the dehydration process can facilitate the inclusion of the resultant food waste products in existing feeding programs of swine [37]. However, Salemdeeb et al. [49] found that the transformation of food waste into dry pig feed has a higher impact on the environment and health mainly due to the higher consumption of fossil fuel required for the dehydration process in comparison with the transformation of food waste into sterilized wet feed. It should be noted that heating of food waste should be carried out at the lowest possible temperatures, and food waste should not be susceptible to oxygen, as well as comply with the recommended storage time. Otherwise, this may lead to lipid peroxidation of the food waste product [38]. Moreover, Cheraghi Saray et al. [71] suggested that probiotic treatment can improve the chemical composition of restaurant waste.

Under this context, a novel approach is implemented in order to transform food waste into animal feed. This approach is implemented with the contribution of the LIFE financial instrument of the European Union under project number LIFE15 ENV/GR/000257. The main expected result of the LIFE-F4F (Food for Feed) project is to deliver a process that allows the safe, economically- and environmentally-viable transformation of food waste from hotels, and the hospitality industry generally, into animal feed. The main principles of this process are to utilize solar energy and use separation schemes at the source of food. This process produces low carbon emissions and is energy efficient due to the fact that solar energy is utilized for pasteurizing and drying of the food waste [72]. Under this context, the first trials for the conversion of hotel food waste to feed product have commenced in Crete, Greece. The dry matter analysis of the dried product showed a dry matter content of 92.74%. Furthermore, on a dry basis, the CP, EE, and crude fiber (CF) contents were 25.62%, 21.57%, and 6.75 %, respectively (unpublished data).

### 3.3. Application in Poultry Nutrition with Emphasis on Fatty Acids Present in Food Waste Material

Another advantage of feeding food waste to monogastric animals, such as poultry and swine, is their ability to incorporate dietary fatty acids in animal products, such as meat and eggs [73]. On the contrary, regarding ruminants, dietary fatty acids are subject to biohydrogenation [73] to a large extent [74] in the rumen [73], a process which results in the production of saturated fatty acids (SFA) with the use of dietary PUFA as a substrate by microbes [75].

In a study conducted by Cho et al. [39], broilers were fed diets containing 0%, 10%, 20%, or 30% dried leftover food (DLF) or 10% DLF and 5% higher protein level (PL), 20% DLF and 10% higher PL or 30% DLF and 15% higher PL than the control diet (Table 2). The DLF used in this study had 20.62% CP, and 9.99% EE. DHA concentration in meat from broiler groups fed DLF was not significantly higher than that of the control group, although it was numerically higher [39]. In the above-mentioned study, EPA concentration of meat from broiler groups fed diets containing 30% DLF, 10% DLF and 5% higher PL, and 30% DLF and 15% higher PL were significantly higher compared to the control group. Cho et al. [39] also found that arachidonic acid content in broiler meat was not significantly different among treatments, although it was numerically higher among groups fed leftover food compared to the control group, except for the group fed 10% DLF and 5% higher PL, which was lower. Another PUFA that Cho et al. [39] examined in broiler meat was linolenic acid, the content of which was numerically higher in all food waste treatments compared to the control group, but it was significantly different in the broiler groups fed diets containing 10% or 30% DLF, and in groups fed diets containing 10% DLF and 5% higher PL, and 20% DLF and 10% higher level PL compared to the control group. As for linoleic acid concentration in broiler meat, it was numerically higher in treatments fed leftover food, but the groups fed diets containing 10% DLF, and 10% DLF and 5% higher PL presented significant differences in comparison to the control group [39]. Furthermore, Cho et al. [39] determined the content of myristic acid, palmitic, and palmitoleic (C16:1) acids in meat. Palmitic acid content did not present significant differences among treatments, while myristic acid content was significantly higher in group fed diet containing 10% DLF and 5% higher PL. Palmitoleic acid (C16:1) content in meat was significantly lower in groups fed diets containing 10% DLF, and DLF and higher PL compared to the control group. The leftover food used in the aforementioned study had 9.99% EE (dry matter 93.70%); however, the fatty acid profile is unknown [39].

On the contrary, Hossein [76] found that incorporation of dehydrated processed restaurant waste at a level above 20% in diet fed to local village chicken breed had a major effect in PUFA content of meat due to the fact that the food waste used in the experiment was high in SFA (Table 2). The restaurant waste had a higher proportion of SFA compared to the diet of the control group, and it also underwent soaking in hot water, and thus, the fat content of restaurant waste was reduced. Regarding the fatty acid content in meat, increased incorporation of dehydrated restaurant waste at a level of 60% in chicken diets lead to decreased PUFA content in chicken meat linearly, and increased SFA content. PUFA content in chicken meat was the highest in the control group (36.05%) and the lowest in the group fed diet containing 60% dehydrated processed restaurant waste (26.82%). Furthermore, n-6 PUFA content was found to be the highest in the control group (34.00%), and the lowest in the group-fed diet containing 60% dehydrated processed restaurant waste (24.35%). The latter group also presented the highest n-3 PUFA content (2.47%), while the control group presented the lowest (2.05%). Moreover, a similar pattern was followed by the PUFA/SFA ratio, which decreased with the increasing incorporation of restaurant waste in chicken diets [76].

The cholesterol of broiler meat has also been determined in the experiment of Cho et al. [39] without significant differences. Most notably, it was reported that broilers fed diets which contained 10% or 20% DLF had numerically higher (101.18 and 102.12 mg/g, respectively) meat cholesterol concentration compared to the control group (92.00 mg/g), which was fed a diet containing 0% leftover food [39].

Kojima [77] carried out an experiment in which laying hens were fed diets containing 0%, 12.5%, 25.0%, or 50.0% of a dehydrated kitchen waste product (Table 2). The kitchen waste used in that study derived from a retirement home. The CP of the dehydrated kitchen waste product was 15.14%, and the EE was 5.33%. Egg yolk from hens fed diet containing 50% kitchen waste had the highest content of PUFA (18.9%) and the lowest of SFA (30.8%) compared to control. Furthermore, C18:1 and C18:3 content in egg yolk tended to increase with increasing incorporation of the kitchen waste product in hen diets.

In summary, the fatty acid composition of poultry meat is dependent on the fatty acid composition of food waste. Therefore, food waste that is derived from food service operations, which use high quality food ingredients, is more likely to provide high-quality meal products. In addition, care should be taken regarding the incorporation of food waste in poultry diet in case that food waste includes residues of fish origin, since it may result in the formation of off-flavors in poultry products. More research on the quality of ingredients, like fatty acids and vitamins, in food waste will enlighten the nutritional value of food waste prior to the incorporation in poultry and swine diets.

### 3.4. Application in Swine Nutrition with Emphasis on Fatty Acids Present in Food Waste Material

Feeding swine with food waste is not a new practice [30]. There is a long history behind the use of food waste in animal nutrition. Livestock animals can be described as “bio-processors” for food not suitable for human consumption or food that was wasted, which can be converted into meat [18]. This practice has been associated with the domestication of swine, as food waste generated by humans may have attracted swine to early settlements [26,78], and it continues to be a common practice in many parts of the world [26]. Although food waste has been an important component in backyard pig production [79], food waste also has a share in the diets of contemporary systems of pig production [49]. In the US, currently in the year 2020, garbage feeding to swine is permitted in 27 states and some other territories [80]. Research on feeding food waste to swine has been carried out for over a century [13].

Swine have the ability to incorporate dietary fatty acids in meat [44,73]. This is supported by a study conducted by Choe et al. [44], in which pigs were fed solely boiled restaurant waste (26.59% CP, 7.33% total lipids) without any other diet in comparison to the control group that was fed a conventional diet (20.21% CP, 15.67% total lipids). More specifically, the experimental group was fed restaurant wastes during the growing (6 wk) and finishing period (12 wk) and then a conventional feed for 4 wk before slaughtering. The control group was fed only the conventional diet that met the recommended nutrient requirements by the National Research Council. Analysis of food waste and conventional feed revealed that in comparison to the conventional diet, food waste had higher PUFA content (25.08% vs. 21.04%) and PUFA/SFA ratio (0.73 vs. 0.57) and lower SFA and MUFA contents [44]. In the above-mentioned study, the fatty acid profile of pork loin and backfat from pigs of the experimental group was positively correlated with the fatty acid profile of the restaurant waste. Pork loin from the group fed restaurant waste presented significantly higher PUFA content (22% vs. 15.21%) and PUFA/SFA ratio compared to the control group, while SFA (37.04% vs. 40.04%) and MUFA (40.96% vs. 44.75%) contents were significantly lower (Table 2). Moreover, pork loin showed a lower n-6/n-3 PUFA ratio in the group fed food waste compared to the control group. Backfat in swine fed restaurant waste showed similar results with those of loin regarding the fatty acid profile with SFA being the exception, which was similar in both experimental group and control groups. Lipid peroxidation was higher in pork loin and backfat of the group fed restaurant waste compared to that of the control group [44]. In such cases where food waste presents a high content of PUFA, the oxidative stability of meat can be improved by adding antioxidants [38], such as α-tocopheryl acetate [81], to swine diets [38].

In the above-mentioned study conducted by Choe et al. [44], some of the PUFA analyzed in pork loin and backfat were EPA, DHA, linoleic acid, linolenic acid, and arachidonic acid. EPA content in pork loin was significantly higher in the food waste fed group (0.44%) compared to the control group (0.09%). Likewise, DHA content in loin was significantly higher in the experimental group (0.71%) in comparison to the control group (0.23%). Similarly, the linoleic acid content in loin was significantly higher in the food waste fed group (17.91%) compared to the control group (12.68%), while linolenic acid presented the same pattern, which was equal to 1.17% in the experimental group and 0.61% in the control group. Arachidonic acid content in loin and backfat was similar in both control and experimental groups. Backfat presented similar results in regard to the previous-mentioned PUFA. EPA content in backfat was significantly higher in the experimental group (0.13%) in comparison to the control group (0.04%), while DHA content was also significantly higher in the food waste fed group (0.52%) compared to the control group (0.17%). In the same way, linoleic acid content in backfat was also significantly higher in the experimental group (18.48%) in comparison to the control group (13.65%), while linolenic acid content presented the same pattern, which was equal to 1.58% in the experimental group and 0.91% in the control group [44].

In an experiment conducted by Kwak and Kang [40], pigs were fed different levels of a food waste mixture (FWM). The FWM was aerobically processed and dried and contained restaurant waste (dry matter 19.1%, CP 22,0%, and crude ash 12.6%), processed broiler litter, and bakery by-product. Pigs were allocated in three different groups and their diets comprised 0% (control group), 25% or 50% FWM on a dry basis and a corn-soy diet. After slaughtering, the fatty acid composition of the longissimus muscle of the pigs was assessed. Feeding of the FWM did not statistically affect the fatty acid components of the meat [40].

From an environmental standpoint, livestock production has been associated with the degradation of the environment since it contributes to global warming aggravation directly and indirectly [82]. Concerning feed production, land expansion in South America for the sake of soybean production leads to deforestation, which accounts for biodiversity decline and high carbon emissions [26]. The transformation of food waste to swine feed has the potential to promote environmental sustainability as it is indicated from the results of two studies. Salemdeeb et al. [49] found that this practice has the least impact on the environment and health compared to anaerobic digestion and composting, and particularly the use of sterilized wet swine feed in comparison with the use of dry feed. In another study, zu Ermgassen et al. [26] stated that the utilization of food waste as pig feed could reduce the land use of pork production in the EU by one-fifth.

## 4. Conclusions

Given that one-third of the total food produced is lost or wasted worldwide and that the demand for animal feed is projected to increase, food waste utilization as animal feed can contribute to the alleviation of food insecurity. Food waste may possess bioactive compounds and nutrients that may favor the transformation into animal feed. On the other side, food waste may exhibit variation in nutrient composition and potential hazards if not treated appropriately. Currently, pig and poultry studies reveal promising results. Further research is needed to be conducted on several aspects, including but not limited to the nutritional composition of different types of food waste, the effect of feeding food waste to pigs and poultry on growth performance, and the quality traits of meat. As the nutritional composition of food waste may vary between regions or countries where food waste is generated, it is important that nutritional analyses provide sufficient data regarding local food waste in order to facilitate the incorporation into animal feed.

In the future, the incorporation of transformed low-cost food waste-derived products into animal diets may provide the possibility to lower production costs, which constitute a large part of the total production costs of poultry and swine. This could be a strong incentive for the stakeholders in the business industry to get involved in the utilization of food waste as animal feed given that quality and safety are guaranteed. Finally, food waste recycling in animal nutrition may contribute to the reduction of the environmental impact and improve livestock production’s environmental footprint and help meet the target of the European Green Deal to make Europe the first climate-neutral continent by 2050.

## Figures and Tables

**Table 1:** A summary of selected studies reporting bioactive compounds and major components of food waste.

Food Waste Type	Origin of Food Waste	Key Findings as Reported by Authors	References
Food service	Restaurant	Average analysis of restaurant waste (RW) from four studies showed range of values CP (15–23%), ash (3–6%), EE (17–24%)	[37]
		RW was ground and had CP (22.0%), ash (12.6%), EE (23.9%) Afterwards, RW was mixed with other feedstuffs and then further processing took place	[40]
		RW was ground in a blade mill, mixed and homogenized, and heat processing took place (65–80 °C, 10–60 min) The nutritional composition of RW was CP (27.5%), Ca (0.42–1.7%), P (0.81–2.07%), EE (28.8%)	[29]
		RW underwent boiling Methionine, cysteine, lysine, P and Ca contents of the RW were quite similar between RW and a conventional diet Threonine and valine were higher in RW Salt content, PUFA, MUFA and SFA contents, and PUFA/SFA ratio were significantly higher in RW The n-6/n-3 fatty acid ratio was significantly lower in RW	[44]
	Hospitality sector	Food waste (FW) from Hotels was dried with the use of solar energy. The nutritional composition of the final product was Dry matter (92.74%), CP (25.62%), EE (21.57%), CF (6.75%)	Unpublished data
		FW contained kitchen and plate waste. FW were minced, pelleted, and dried FW contained CP (18–20%), ash (5–6%), salt content (2.0–2.5%), Ca (0.5–0.8%), P (0.3–0.8%), EE (24–26%) Lysine, methionine, threonine, and tryptophan contents were higher compared to corn, but lower in comparison with soybean meal Afterwards, FW were blended with a dry feedstock	[16]
	Restaurant and hotel	Leftover food was minced, heated and dried in hot air oven at 85 °C for 4 h CP (31.3%), ash (14.75%), EE (26.08%)	[42]
Food service and Households	Restaurant and apartment complex areas	FW was dried in a drum type dryer at 115 ± 2 °C CP (25%), salt content (3.28%), EE (17.3%) The majority of the essential amino acids, such as methionine and lysine, were considerably lower in quantity than that of a corn and soybean mix (60%:40% ratio)	[41]
	Commercial and residential locations	FW contained CP (27.6%), ash (14.56%), Ca (1.09–1.25%), P (0.16–0.30%), K (0.56–0.76%), Mg (0.1–0.2%), EE (9.12%), oleic acid (30.63% of EE), linoleic acid (25.5% of EE), linolenic acid (3.03% of EE), and had PUFA/SFA ratio (0.78), n-6/n-3 fatty acid ratio (7.94)	[28]
Institutional	University dining hall	FW was dried in a forced-air oven at 60 °C for 72 h. Samples were ground and mixed FW contained CP (18.9%), ash (5.01%), Ca (0.04–0.46%), P (0.23–0.37%), EE (13.58%), arachidonic acid (0.20% of EE), linoleic acid (29.31% of EE), linolenic acid (3.82% of EE)	[38]
Food services Institutional, Military, and Municipal	Hotel and restaurant etc.	Thiamine and niacin concentrations of cooked food waste were adequate to meet the nutritional requirements for swine, while the pantothenic acid concentration was deficient	[46]
Type unknown	Origin unknown	Leftover food was processed using fluidized bed dry method. Leftover food contained CP (22%) and EE (10.66%)	[39]

Parameters are reported as mean and on a dry matter basis; Ca= calcium; CF = crude fiber; CP = crude protein; EE = ether extract; FW = food waste; MUFA = monounsaturated fatty acid; P =phosphorus; PUFA = polyunsaturated fatty acid; RW = restaurant waste SFA = saturated fatty acid.

**Table 2:** A summary of selected studies reporting effects of adding food waste to poultry and pig diets.

Animal Model	Study Design	Key Findings as Reported by Authors	Reference
Broiler (Ross)	Diets contained 0%, 10%, 20%, or 30% dried leftover food (DLF) or 10% DLF and 5% higher protein level (PL), 20% DLF and 10% higher PL or 30% DLF and 15% higher PL than control diet	DLF contained 20.62% CP, and 9.99% EE DHA content was numerically higher in meat of DLF groups, but was not significantly different EPA content in meat was significantly higher in meat of the 30% DLF, 10% DLF and 5% higher PL, and 30% DLF and 15% higher PL groups compared to the control group Linolenic acid content in meat was numerically higher in DLF groups, though significantly higher in 10% and 30% DLF treatments, and in 10% DLF and 5% higher PL, and 20% DLF and 10% higher PL groups compared to the control group Linoleic acid content in meat was numerically higher in DLF groups, though significantly higher in 10% DLF, and 10% DLF and 5% higher PL groups compared to the control group Myristic acid content in meat was significantly higher in 10% DLF and 5% higher PL group Palmitic acid content in meat presented no significant differences among treatments Palmitoleic acid (C16:1) content in meat was significantly lower in 10% DLF, and DLF and higher PL groups compared to the control group Arachidonic acid content in broiler meat showed no significant differences among treatments. Cholesterol concentration in broiler meat was numerically higher in groups fed diets containing 10% or 20% DLF (101.18 and 102.12 mg/g, respectively) compared to the control group (92 mg/g)	[39]
Free range village chickens	Diets contained dehydrated restaurant waste (RW) at 0%, 20%, 40% or 60% level	RW had a higher proportion of SFA than the diet of the control group SFA content in meat was significantly higher in groups fed RW PUFA content in meat decreased linearly with increasing inclusion of RW in the diet PUFA/SFA ratio decreased with increasing incorporation of RW in diets The minimum PUFA/SFA ratio was found in group fed diet containing 60% RW, which increased with decreasing inclusion of RW in the diet n-6 fatty acid content decreased with increasing inclusion of RW in diet, while the n-3 fatty acid content increased	[76]
Laying hens	Diets contained 0%, 12.5%, 25% or 50% dehydrated kitchen waste product	The dehydrated kitchen waste product had 15.14% CP, and 5.33% EE PUFA content was the highest in egg yolk from hens fed diet containing 50% kitchen waste, while SFA was the lowest compared to control C18:1 and C18:3 content in egg yolk tended to elevate with increasing incorporation of kitchen waste in diets.	[77]
Swine	Control group was fed a conventional diet, and experimental group was fed solely boiled restaurant waste during the growing (6 wk) and finishing period (12 wk) and then the conventional feed for 4 wk before slaughtering	CP and total lipids of RW were 26.59% and 7.33%, respectively CP and total lipids of the conventional diet were 20.21% and 15.67%, respectively PUFA content in RW vs. control feed was 25.08% vs. 21.04% while PUFA/SFA ratio was 0.73 vs. 0.57 Positive correlation of fatty acid profile between RW and pork loin, and backfat PUFA content in pork loin compared to the control group was 22% vs. 15.21% and PUFA/SFA ratio 0.6 vs 0.38 was significantly higher, while SFA (37.04% vs. 40.04%) and monounsaturated fatty acid content (MUFA, 40.96% vs. 44.75%) was significantly lower in the experimental group Back fat in swine fed RW showed similar results with those of loin regarding the fatty acid profile with SFA being the exception Lipid peroxidation of pork loin was higher in the group fed RW In comparison to control, pork loin of swine fed RW had higher concentration of EPA (0.44% vs. 0.09%), DHA (0.71% vs. 0.23%), linoleic acid (17.91% vs. 12.68%), and linolenic acid (1.17% vs. 0.61%) EPA (0.13% vs. 0.04%), DHA (0.52% vs. 0.17%), linoleic acid (18.48% vs. 13.65%), and linolenic acid (1.58% vs. 0.91%) content in backfat was significantly higher than that of the control group Arachidonic acid content in loin and backfat was similar in both the control and experimental groups	[44]
Swine	Diets contained 0%, 25%, or 50% food waste mixture and a corn-soy diet	Percentage of total SFA and USFA, MUFA/SFA and PUFA/SFA ratios of longissimus muscle were not affected by the incorporation of the food waste mixture	[40]

CF = crude fiber; CP = crude protein; DLF = dried leftover food; DHA = Docosahexaenoic acid; EE = ether extract; EPA = Eicosapentaenoic acid; MUFA = monounsaturated fatty acid; PL = protein level; PUFA = polyunsaturated fatty acid; RW = restaurant waste; SFA = saturated fatty acid; USFA = unsaturated fatty acid
